# Prevalence and associated factors of depression among Korean adolescents

**DOI:** 10.1371/journal.pone.0223176

**Published:** 2019-10-16

**Authors:** Je-Yeon Yun, Halin Chung, Jin-ah Sim, Young Ho Yun

**Affiliations:** 1 Seoul National University Hospital, Seoul, Republic of Korea; 2 Yeongeon Student Support Center, Seoul National University College of Medicine, Seoul, Republic of Korea; 3 Department of Biomedical Science, Seoul National University College of Medicine, Seoul, Republic of Korea; 4 Department of Family Medicine, Seoul National University Hospital, Seoul, Republic of Korea; 5 Department of Biomedical Informatics, Seoul National University College of Medicine, Seoul, Republic of Korea; University of Ghana, GHANA

## Abstract

This study aimed to identify factors significantly associated with recent depressive mood with respect to health-related behavioral patterns at the individual level, perceived safety in the school environment, and willingness to share concerns with family and social networks. Self-reported responses to questions regarding recent feelings of depression, health-related behaviors in physical, psychological, and spiritual subdomains, school refusal and perceived safety at school, and perceived social support were obtained from 1,991 in-school adolescents (mean [SD] age = 15.3 [1.7] years; male/female = 936/1055). Multivariate logistic regression analyses were used to identify explanatory factors significantly associated with recent depression, defined as feelings of sadness or hopelessness for more than 2 weeks (during the last 12 months) that interfered with everyday functioning. Of the 1,991 students, 271 (13.6%) reported recent depression. Multivariate logistic regression analyses revealed higher odds of recent depression in adolescents with frequent thoughts of school refusal (odds ratio [95% confidence interval] = 3.25 [2.44–4.32]) and those who engaged in regular physical exercise (1.57 [1.19–2.07]), whereas a positive mindset (0.65 [0.49–0.86]), perceived safety at school (0.62 [0.47–0.82]), and perceived social support from one’s mother (0.54 [0.40–0.72]) were associated with lower odds of recent depression. Taken together, our findings suggest that parents and teachers should talk regularly with adolescents about recent life (dis)satisfaction and stressors, particularly when they report frequent thoughts of school refusal. Perceived social support would increase perceived safety on school grounds and make it easier for teenagers to share their concerns with parents, thereby reducing the risk for depressive symptoms. School-based programs that promote a positive mindset would be helpful in preparing students for the challenges of adulthood.

## Introduction

### Depression in adolescence

Mental health problems including internalizing psychopathologies such as depressive mood and anxiety may develop during adolescence [1]. Importantly, depression during adolescence could affect the trajectory of personality development and academic achievement, which in turn may impair social functioning during adulthood [2, 3]. Furthermore, the lack of appropriate treatment for depression and suicidality in adolescents and young adults increases the likelihood of mood disorders later in life [4, 5]. However, parents and schoolteachers often do not recognize the early signs of depression in adolescents and the need for stress management in daily activities [6, 7].

### Behavioral routine of daily living and depression

Previous studies have reported correlations between increased risk of depression and substance use (i.e., alcohol, tobacco, and cannabis) [8], deliberate food restriction for weight control [9], and obesity. By contrast, protective factors against depression include daily consumption of vegetables and fruit [10], regular sleep, and frequent participation in sports and high-intensity physical activity [11]. Furthermore, a well-balanced lifestyle that enables work–life balance [12], prosocial activities focused on helping others [13], and religious activities to clarify the meaning of life [14] may be helpful in reducing the risks of burnout and depression.

### Stress-related cognitive style and depression

Previous studies have reported positive correlations between increased risk for adolescent depression and the use of avoidant–maladaptive coping strategies (e.g., worry, self-blame, distraction, disengagement, tension reduction, and escape from negative emotions through substance misuse) [8, 15] and emotional–impulsive decision making in the face of stressful daily life events. By contrast, modifiable protective factors against depression include lifestyle factors such as prosocial involvement in a family context [16] and adaptive coping strategies (e.g., focusing on the positive, problem solving, and seeking social support) that stem from a positive mindset [8, 15]. Notably, many previous studies have posited the importance of a positive mindset (e.g., self-efficacy [17, 18], optimism [19], acceptance [20], resilience [21], and gratitude [22, 23]) in helping patients and caregivers to cope with debilitating medical illnesses such as chronic obstructive pulmonary disease, Parkinson’s disease, and multiple sclerosis [19–21, 24, 25]; in fostering healthy development in children facing physical disability after trauma [17]; and in enhancing academic achievement [25, 26], elite sports performance [18], and creative art work [27]. Moreover, the presence of a positive mindset, such as that reflected in resilience, was associated with greater positive affect and reduced depression in community-dwelling middle-aged adults [28].

### School refusal and perceived safety at school in adolescence

Given the amount of time adolescents spend at school, the school environment and school-related activities may be the greatest source of protection against, or the greatest source of, distress for adolescents [29, 30]. Indeed, school refusal (self-motivated refusal to attend school and/or difficulty remaining in class for the full day) [31] might not be simply a maladaptive behavior [32]. Rather, it may often be a warning sign of severe distress and negative emotional states including anxiety and depressive mood that markedly impair social and daily functioning [33]. Better understanding of the importance of school refusal as a possible warning sign of depressive symptoms and clarification of lifestyle- or environment-related factors that can affect the risk of adolescent depression, will enable parents and teachers to recognize emotional distress and help adolescents who refuse to attend school.

### Study aim and hypothesis

The long list of behavioral factors that positively or negatively affect the risk for depression in adolescents shown above may leave parents, teachers, and adolescents themselves confused about the best first steps to take in recognizing emotional distress [7, 34] and strengthening stress resilience [35]. Accordingly, the current study used self-reports from school-attending adolescents (N = 1,991) to identify the most significant factors associated with feelings of sadness or hopelessness (for more than 2 weeks in the last 12 months) that interfered with daily life functioning. We hypothesized that the odds of recent experiences of depressive mood among in-school adolescents may be associated with distress about school attendance [33]; perceived level of safety at school [36]; one’s own lifestyle related to physical [37, 38], psychological [39–42], and spiritual [43, 44] sub-domains of health; and perceived social support that enables adolescents to share their concerns with others and get help [45–47].

## Materials and methods

### Participants

Data were collected between August 2014 and January 2015 in a cross-sectional survey for the validation and field-testing of the Korean version of the School Health Score Card (SHSC) [48]. With cooperation of the Korean Association of Secondary Education Principals, 30 schools (15 middle schools and 15 high schools) from a diverse range of provinces in the Republic of Korea participated in the study. Following a detailed description of the study purpose and procedure, 2,800 students provided informed consent and completed a five-part self-report questionnaire (S1 and S2 Texts): Part 1 assessed level of satisfaction with one’s own health condition and health-related behavior, Part 2 assessed physical and behavioral health-related factors and perceived safety of the school environment, Part 3 assessed psychological health-related factors, Part 4 assessed social health-related factors (people to discuss concerns with), and Part 5 assessed awareness and prior experience with health-enhancement programs in school (see ‘Measures’ below for detailed information on the items included in Parts 1–4). Our study included the subset of participants who responded to all of the items in the Parts 1–4, (Part 5 data were not included in our study) without omission or error responses. Therefore, the final analyses included 1,991 students from 30 schools (mean [SD] age, 15.3 [1.7] years; male/female, 936/1055). The Institutional Review Board of Seoul National University College of Medicine approved this study (IRB No. E-1407-127-597). Informed consent was obtained from all participants after the procedure was fully explained. As this was a minimal-risk study, the requirement for written consent from the individual participants was waived by the board. All procedures were performed in accordance with the ethical standards of the Seoul National University Hospital Institutional Review Board on Human Experimentation and the Helsinki Declaration of 1975, as revised in 2008.

### Measures

Data were obtained using self-report questionnaires (please refer to Supporting Information) completed by 1,991 adolescent students; 17 items regarding health-related behaviors (7 items) [49, 50], school refusal and perceived safety at school (4 items) [51], and perceived social support (6 items) [51] were used to identify the most strongly associated factors that could explain recent feelings of depression, defined in terms of a ‘Yes’ (= depressed) or ‘No’ (= non-depressed) response to the item “Have you had feelings of sadness or hopelessness for more than 2 weeks (in the last 12 months) that interfered with your daily functioning?”] [52–54].

First, responses to seven items about health-related behaviors [49, 50], categorized by physical [1) regular physical exercise of moderate intensity and >150 min per week, 2) healthy eating habits, and 3) lifestyle balanced between study and rest], psychological [4) positive mindset and 5) proactive lifestyle], and spiritual [6) make time for helping others and 7) maintain faith and religious activities] subdomains, were retrieved. The regular physical exercise question required a ‘Yes’ (0) or ‘No’ (1) binary response. The ordinal responses to the remaining questions were converted into binary values (‘Have been practicing more than 6 months’ [1] and ‘Have been practicing less than 6 months’ [2] were converted to ‘Have been practicing’ [0]; and ‘Planning to start within 1 month’ [3], ‘Planning to start within 6 months’ [4], and ‘No plan to practice in the future’ [5] were converted to ‘Not started yet’ [1]), and these later underwent stepwise multiple linear regression.

Second, four items addressing school refusal/perceived safety at school [51] were as follows: 1) being able to ask for help when needed, 2) awareness of risky areas in the school zone, and belief that the school grounds are free from 3) bars and 4) gambling venues. The ordinal responses were converted into binary values for use in a subsequent stepwise multiple linear regression analyses (‘Do not know’ [1], ‘Totally false’ [2], and ‘False’ [3] became ‘False’ [1]; and ‘True’ [4] and ‘Totally true’ [5] became ‘True’ [0]). One variable selected from Part 4 (psychological health-related factors) that concerned frequent thoughts of school refusal was converted from ordinal responses to binary values (‘Totally disagree’ [1] and ‘Slightly true’ [2] were re-coded as ‘No’ [0]; and ‘Agree‘[3] and ‘Strongly agree’ [4] were re-coded as ‘Yes’ [1]).

Third, six items regarding perceived social support [51] assessed respondents’ perception of the ease of being able to discuss their concerns with 1) their father, 2) their mother, 3) siblings, 4) friends of the same sex, 5) friends of the opposite sex, or 6) teachers at school. The initial ordinal responses were converted into binary values (‘Not applicable’ [1], ‘Not possible’ [2], and ‘No’ [3] became ‘No’ [1]; and ‘Yes’ [4] and ‘Very much so’ [5] were re-coded as ‘Yes’ [0]) for inclusion as candidate explanatory variables in subsequent multiple logistic regression analyses.

### Statistical analyses

Based on their response to the question about feelings of sadness or hopelessness for more than 2 weeks, participants were classified as ‘depressed’ (*n =* 271) or ‘non-depressed’ (*n =* 1,720), and these two groups were compared in terms of demographic characteristics (age and body mass index) using independent *t*-tests (*P <* (0.05/4) = 0.013), and associations between group membership (depressed versus non-depressed) and sex (male or female) or level of school attendance (middle school or high school) were calculated using chi-square tests (*P <* (0.05/4) = 0.013). Simple logistic regression analyses were used to calculate the associations of the 17 items (see the ‘Measures’ section above) addressing health-related behaviors (7 items) [49, 50], school refusal/perceived safety at school (4 items) [51], and perceived social support (6 items) [51] with recent experiences of depression [52–54] (**Table 1**). Items that showed significant associations with recent depressive feelings (P < 0.05) were used as candidate explanatory variables in the subsequent multivariate logistic regression analyses (with variable selection methods for ‘forward: LR’ and ‘backward: LR’). Adjusted ORs of each final explanatory variable for recent depression were estimated from the final multivariate regression model. All statistical analyses were performed using IBM SPSS Statistics version 24 (IBM Corp., Armonk, NY, USA).

**Table 1:** Estimated effects of demographic and clinical characteristics on experiencing depressive mood (n = 1,991).

Depressive = Feelings of sadness or hopelessness for more than 2 weeks (in the last 12 months) that interfered with daily life.	Depressed (n = 271)	Non-depressed (n = 1,720)	Unadjusted OR (95% CI)/ χ^2^ score	*P*-value (Wald test/t-test)	Use in the multivariate logistic regression (forward LR/backward LR)
Age, mean (SE)	15.5 (1.6)	15.2 (1.7)	t(1989) = 2.13	0.033	NA
Sex (%), M/F	117/154	819/901	χ^2^(1) = 1.86	0.173	NA
Body mass index	21.1 (3.7)	20.7 (3.1)	t(334.46) = 1.44	0.15	NA
Level of school, middle school/high school	106/165	792/928	χ^2^(1) = 4.54	0.033	NA
***Part 1*: *Health-related behaviors*: *physical*, *psychological*, *and spiritual***					
Physical: regular physical exercise (moderate intensity and >150 min/week) [Y/N]	124/147	678/1042	1.296 [1.002–1.678]	0.048	O
Physical: healthy eating habits [Y/N]	124/147	944/776	0.693 [0.536–0.897]	0.005	O
Physical: lifestyle balanced between study and rest [Y/N]	108/163	846/874	0.685 [0.527–0.889]	0.004	O
Psychological: positive mindset [Y/N]	141/130	1163/557	0.519 [0.401–0.673]	<0.001	O
Psychological: proactive lifestyle [Y/N]	132/139	1042/678	0.618 [0.478–0.799]	<0.001	O
Spiritual: make time for helping others [Y/N]	115/156	777/943	0.895 [0.691–1.159]	0.4	×
Spiritual: maintain faith and religious activities [Y/N]	91/180	567/1153	1.028 [0.784–1.349]	0.842	×
***Part 2*: *School refusal/perceived safety at school***					
Frequent thoughts of school refusal [Y/N]	125/146	296/1424	4.119 [3.145–5.394]	<0.001	O
Perceived level of safety on the school grounds: able to ask for help [Y/N]	148/123	1280/440	0.414 [0.318–0.538]	<0.001	O
Perceived level of safety on the school grounds: have information about risky areas [Y/N]	122/149	838/882	0.862 [0.666–1.115]	0.257	×
Perceived level of safety on the school grounds: free from bars and gambling venues [Y/N]	143/128	1099/621	0.631 [0.488–0.817]	<0.001	O
***Part 3*: *Perceived social support (people with whom concerns are discussed)***					
Perceived availability of father for discussing concerns [Y/N]	136/135	1129/591	0.527 [0.407–0.683]	<0.001	O
Perceived availability of mother for discussing concerns [Y/N]	178/93	1432/288	0.385 [0.291–0.510]	<0.001	O
Perceived availability of siblings for discussing concerns [Y/N]	138/133	1030/690	0.695 [0.538–0.899]	0.006	O
Perceived availability of friends of the same sex for discussing concerns [Y/N]	216/55	1515/205	0.531 [0.382–0.739]	<0.001	O
Perceived availability of friends of the opposite sex for discussing concerns [Y/N]	124/147	780/940	1.017 [0.786–1.315]	0.9	×
Perceived availability of schoolteachers for discussing concerns [Y/N]	115/156	994/726	0.538 [0.415–0.698]	<0.001	O

## Results

Of the 1,991 adolescents who participated in the study, 271 (13.6%) reported recent (in the last 12 months) sadness or hopelessness for more than 2 weeks that interfered with daily life functioning. We found no significant differences in age, sex, body mass index, or school level (middle school vs. high school) between depressed (*n =* 271) and non-depressed (*n =* 1,720) adolescents (*P <* (0.05/4) = 0.013; **Table 1**).

Simple logistic regression analyses revealed that the odds of recent feelings of depression were increased in adolescents with frequent thoughts of school refusal (crude OR [95% confidence interval] = 4.12 [3.15–5.39]) and in those who engaged in regular physical exercise (1.30 [1.00–1.68]). By contrast, physical health-related behaviors such as healthy eating habits (0.69 [0.54–0.90]) and a lifestyle balanced between study and rest (0.69 [0.53–0.90]); psychological health-related factors including a positive mindset (0.52 [0.40–0.67]) and proactive lifestyle (0.62 [0.48–0.80]); perceived school zone safety, including the ability to ask for help (0.42 [0.32–0.54]) and a school environment free from bars and gambling venues (0.63 [0.49–0.82]); and perceived availability of father (0.53 [0.41–0.68]), mother (0.39 [0.29–0.51]), siblings (0.70 [0.54–0.90]), friends of the same sex (0.53 [0.38–0.74]), or school teachers (0.54 [0.42–0.70]) were associated with a decreased risk for recent experiences of depression (*P <* 0.05; **Table 1**).

Multivariate logistic regression analyses with these 14 candidate variables (obtained from simple logistic regression analyses with *P <* 0.05; refer to the paragraph above and **Table 1**) revealed an increased risk of recent depressive feelings in adolescents who reported frequent thoughts of school refusal (adjusted OR = 3.25 [2.44–4.32]) and in those who engaged in regular physical exercise (1.57 [1.19–2.07]), whereas the health-related behaviors of positive mindset (0.65 [0.49–0.86]), perceived school zone safety including the ability to ask for help (0.62 [0.47–0.82]), and perceived availability of mother for support (0.54 [0.40–0.72]) were associated with a decreased risk for recent depression (**Table 2**). The multivariate logistic regression models were the same regardless of the variable selection method applied (forward LR or backward LR) and had significant goodness of fit (Hosmer–Lemeshow test = 0.394 > 0.05; percentage of correct classification = 86.4% >70%; area under the receiver operating characteristic curve for classifying group membership = 0.71 [0.68–0.75]).

**Table 2:** Multivariate logistic regression (with forward LR/backward LR applied for variable selection from candidate variables in Table 1): Estimated effects of health-related behaviors, school refusal/perceived safety at school, and perceived social support on recent depression.

Depression = Feelings of sadness or hopelessness for more than 2 weeks (in the last 12 months) that interfered with daily life.	b	Adjusted OR (95% CI) based on multivariate logistic regression	*P*-value (Wald test)
***Part 1*: *Health-related behaviors*: *physical*, *psychological*, *and spiritual***			
Physical: regular physical exercise (moderate intensity and >150 min/week)	0.449	1.566 (1.187–2.067)	0.002
Psychological: positive mindset	−0.435	0.648 (0.489–0.858)	0.002
***Part 2*: : *School refusal/perceived safety at school***			
Frequent thoughts of school refusal	1.179	3.250 (2.444–4.324)	<0.001
Perceived level of safety on the school grounds: able to ask for help	−0.479	0.620 (0.466–0.824)	0.001
***Part 3*: *Perceived social support (people with whom concerns are discussed)***			
Perceived availability of mother for discussing concerns	−0.624	0.536 (0.397–0.724)	<0.001

## Discussion

### Summary

We used a self-report questionnaire dataset obtained from school-attending adolescents (*N =* 1,991) to identify factors significantly associated with the recent experience of depressive mood that interfered with daily living. Of the 1,991 students, 271 (13.6%) answered ‘yes’ to a single item asking about feelings of sadness or hopelessness for more than 2 weeks (in last 12 months) that interfered with everyday functioning. Although a diagnosis of depression cannot be based on this single item but must be confirmed through clinical evaluation by a certified psychiatrist, use of this single item might be more suitable for initial detection of recent experiences of depression in a community population [52–55]. Multivariate logistic regression analyses revealed higher odds of recent depression in adolescents with frequent thoughts of school refusal (odds ratio [95% confidence interval] = 3.25 [2.44–4.32]) and in those who engaged in regular physical exercise (1.57 [1.19–2.07]), whereas a positive mindset (0.65 [0.49–0.86]), perceived school zone safety (0.62 [0.47–0.82]), and perceived social support from one’s mother (0.54 [0.40–0.72]) were associated with lower odds of recent depression.

### School refusal: A possible indicator of adolescent depression

The most significant finding of our study is that frequent thoughts of school refusal are critical warning signs of adolescent depression. School refusal (self-motivated refusal to attend school and/or difficulty remaining in class for the full day) may be associated with loss of motivation for school attendance, the absence of social connectedness, sustained distress at school, social phobia or separation anxiety, and depressive mood [31–33, 56, 57]. Depressed individuals whose condition has not been identified likely suffer from diverse symptomatology and more severe functional impairment than their peers who have been diagnosed and are under proper psychiatric treatment [58]. Therefore, attentive detection of warning signs for depression, such as school refusal and insomnia, at an earlier stage, followed by referral to a physician for proper clinical treatment and efforts to identify and reduce the principal stressors, such as peer bullying at school, might be critical [58, 59].

### More regular physical activity in adolescent depression

In the current study, higher odds for recent depression were shown among school-attending adolescents who participated in regular physical activity of moderate intensity. By contrast, other cross-sectional studies have reported an association between regular physical exercise and fewer depressive symptoms in adults [60, 61] and reduced risk of self-mutilating behavior in adolescents [62]. Furthermore, confounding clinical features such as the individual’s tendency toward anxiety [63], the degree of self-efficacy [64], the compulsive nature of exercise [65], pressure for better performance among elite sports players [66], and the nature of sedentary behaviors (passive vs. mentally active) in daily living [67] could affect the pattern of the relationship between regular physical exercise and depressive symptoms. Physical activity and adolescent mental health interact in a bidirectional way [68]. Previous studies that applied physical activity programs as an intervention for adolescents have mainly examined the effects on weight control and did not focus on the impact of regular physical exercise on mental health [69]. Thus, further longitudinal studies are required to elucidate the profile and direction of influences between regular physical activity and depression in school-attending adolescents.

### Positive mindset as a stress resilience factor in adolescence

A positive mindset could be defined as a cognitive–emotional–behavioral style that approaches stressors in one’s life with an optimistic outlook, accepting reality as it is and making the most of the potentially bad situations with resilience, self-esteem, and self-efficacy [19, 70, 71]. Our finding that a positive mindset was associated with reduced risk for depressive mood in school-attending adolescents is consistent with a recent large-population study of more than 10,000 participants worldwide, which found that the strength of stress resilience (one’s ability to positively adapt and manage stressful experiences using diverse coping strategies) was negatively correlated with depressive mood severity [72]. Another study found that stress resilience mediated the effects of social support on depressive mood [73]. A balanced attributional style for positive events may underlie the lower risk for depressive mood in middle school students who tend to have greater self-esteem than in high school students [74]. Moreover, well-developed stress resilience in late adolescence is associated with a reduced risk for bipolar disorder in adulthood [75]. Furthermore, a study in high-risk adolescents and young adults with depression and suicidal ideation found that increased use of a positive mindset, such as positive reframing, and decreased use of negative cognitive styles, such as self-blame and disengagement, lowered the risk for suicide at baseline and 4 months after the intervention [40]. Because stress resilience can be modified, an ongoing randomized controlled study to modify negative cognitive style by disrupting a selective bias toward negative information and thoughts is needed to investigate whether enhanced stress resilience is a cost-effective [76] method for reducing depressive symptoms in adolescents [77].

### Perceived social support and sharing concerns with parents

We found that the degree of perceived ease in sharing one’s concerns with their mother was significantly associated with fewer reports of depressive mood in school-attending adolescents. Our finding is consistent with that of a previous study, which found the quality of communication with a parent (mother for girls and father for boys) had effects on depression and suicidal ideation in adolescents [78]. Similarly, a recent longitudinal investigation of the social interaction–depressive mood association using growth curve modeling found an association between adolescent–parent communication (with father for boys and with mother for girls) and a decrease in depressive symptoms as the adolescents reached early adulthood [79]. Moreover, female willingness to communicate with their parents is markedly related to sexual behavior in girls and the use of emergency contraceptive pills during adolescence [80]. With regard to the treatment of depressive symptoms, sustained emotional warmth from both parents has been shown to reduce significantly the severity of depressive symptoms experienced by male and female children and adolescents [81]. School-based programs targeting parent–student dyads or triads that focus on parental stress management skills [82] and emotionally attuned communication based on cognitive empathy [83, 84], offered during school vacations or on weekends, may help promote meaningful adolescent–parent communication.

### Study limitations

Our study had several limitations. First, the cross-sectional study design does not allow causal inferences to be made between the intensity of perceived depressive mood and environmental, interpersonal, and individual cognitive factors. Second, use of one self-reporting item cannot be regarded as an equal substitute for clinical diagnosis based on a face-to-face interview by a trained physician. Third, as our study population consisted of middle school and high school students, we urge caution in generalizing our findings to adolescents who do not attend school.

## Conclusions

Taken together, our findings suggest that parents and schoolteachers should talk regularly with adolescents about recent life (dis)satisfaction and stressors, particularly when teens report frequent thoughts of school refusal. Perceived social support would increase perceived safety on school grounds (i.e., students can find teachers and get help) and make it easier for young people to share their concerns with parents, thereby reducing the risk for depressive symptoms. School-based programs that promote a positive mindset would be helpful in preparing students for the diverse challenges of adulthood.

## Supporting information

S1 Text Self-reporting questionnaire (English version). (DOC)

S2 Text Self-reporting questionnaire (Korean version). (DOC)
