# Supplementary material for: Prevalence and associated factors of depression among Korean adolescents
Source: PLoS One. 2019 Oct 16;14(10):e0223176. doi: 10.1371/journal.pone.0223176 (PMC6795486; doi:10.1371/journal.pone.0223176)
Supplement: S1 Text — (DOC) [file pone.0223176.s001.doc]

**S1 Text. Self-reporting Questionnaire (English Version)**

# Part 0. Recent Experiences of Depressed Mood

Q0. During the past year, have you felt sad or depressed enough that you had trouble in your daily life for a period of 2 weeks or more?

1. Yes ② No

# Part1. Health-related Behaviors: Physical, Psychological, and Spiritual

Q1. Do you engage in physical exercise of moderate intensity for at least 150 minutes per week?

1. Yes ② No

Q2-3. How much do you practice the following activities to manage your physical health?

|  | have been practicing for more than 6 months | have been practicing for less than 6 months | planning to start within 1 month | planning to start within 6 months | no plan for practicing this in the future |
| --- | --- | --- | --- | --- | --- |
| Q2. healthy eating habits | ① | ② | ③ | ④ | ⑤ |
| Q3. self-sustainable lifestyle | ① | ② | ③ | ④ | ⑤ |

Q4-5. How much do you practice the following activities to manage your mental health?

|  | have been practicing for more than 6 months | have been practicing for less than 6 months | planning to start within 1 month | planning to start within 6 months | no plan for practicing this in the future |
| --- | --- | --- | --- | --- | --- |
| Q4. positive mindset | ① | ② | ③ | ④ | ⑤ |
| Q5. proactive lifestyle | ① | ② | ③ | ④ | ⑤ |

Q6-7. How much do you practice the following activities to manage your spiritual health?

|  | have been practicing for more than 6 months | have been practicing for less than 6 months | planning to start within 1 month | planning to start within 6 months | no plan for practicing this in the future |
| --- | --- | --- | --- | --- | --- |
| Q6. make time for helping others | ① | ② | ③ | ④ | ⑤ |
| Q7. maintain faith  and religious activities | ① | ② | ③ | ④ | ⑤ |

# Part 2. School Refusal/Perceived Safety at School

Q8. Read each item carefully and check the response that best describes you.

|  | totally disagree | slightly true | quite agree | very much |
| --- | --- | --- | --- | --- |
| I frequently don’t want to go to school | ① | ② | ③ | ④ |

Q9-11. Check the description of the environmental safety of the school zone that best describes your situation.

|  | do not know | totally false | false | true | totally true |
| --- | --- | --- | --- | --- | --- |
| Q9. I can ask anyone for help when needed | ① | ② | ③ | ④ | ⑤ |
| Q10. I know the specific risky spots in the school zone | ① | ② | ③ | ④ | ⑤ |
| Q11. I believe the school zone is safe from entertainment spots such as bars and gambling rooms | ① | ② | ③ | ④ | ⑤ |

# Part 3. Perceived Social Support

Q12-17. Can you discuss your concerns with the following people?

|  | n/a | not possible  at all | no | yes | very much |
| --- | --- | --- | --- | --- | --- |
| Q12. father | ① | ② | ③ | ④ | ⑤ |
| Q13. mother | ① | ② | ③ | ④ | ⑤ |
| Q14. siblings | ① | ② | ③ | ④ | ⑤ |
| Q15. friends of the same sex | ① | ② | ③ | ④ | ⑤ |
| Q16. friends of the opposite sex | ① | ② | ③ | ④ | ⑤ |
| Q17. teachers at school | ① | ② | ③ | ④ | ⑤ |
