# Supplementary material for: Prevalence and associated factors of depression among Korean adolescents
Source: PLoS One. 2019 Oct 16;14(10):e0223176. doi: 10.1371/journal.pone.0223176 (PMC6795486; doi:10.1371/journal.pone.0223176)
Supplement: S2 Text — (DOC) [file pone.0223176.s002.doc]

**S2 Text. Self-reporting Questionnaire (Korean Version)**

# Part 0. 최근 1년간 우울경험

문0. 최근 1년 동안 연속적으로 2주 이상 일상생활에 지장이 있을 정도로 슬프거나 절망감 등을 느낀 적이 있습니까?

① 예 ② 아니오

# Part 1. 건강관련 행동- 신체적, 심리적, 영적 측면

문1. (운동) 본인은 중등도 (운동 시 땀이 나면서 숨이 차지만 말을 할 수 있는 정도) 이상의 운동 및 신체활동을 일주일에 150분 이상으로 하십니까?

① 예 ② 아니오

문2-3. 본인은 현재 신체적 건강관리를 위해 다음 활동을 얼마나 하고 계십니까?

|  | 실천한지  6개월 넘음 | 실천한지  6개월 미만 | 1개월 이내 실천할 예정 | 6개월 이내 실천할 예정 | 실천할 생각 없음 |
| --- | --- | --- | --- | --- | --- |
| 2) 건강한 음식 바르게 먹기 | ① | ② | ③ | ④ | ⑤ |
| 3) 나에게 맞는 생활하기  (과도하지 않은 학습, 충분한 수면) | ① | ② | ③ | ④ | ⑤ |

문4-5. 본인은 현재 정신적 건강관리를 위해 다음 활동을 얼마나 하고 계십니까?

|  | 실천한지  6개월 넘음 | 실천한지  6개월 미만 | 1개월 이내 실천할 예정 | 6개월 이내 실천할 예정 | 실천할 생각 없음 |
| --- | --- | --- | --- | --- | --- |
| 4) 긍정적인 마음 갖기 | ① | ② | ③ | ④ | ⑤ |
| 5) 적극적인 삶 살기 | ① | ② | ③ | ④ | ⑤ |

문6-7. 본인은 현재 영적 건강관리를 위해 다음 활동을 얼마나 하고 계십니까?

|  | 실천한지  6개월 넘음 | 실천한지  6개월 미만 | 1개월 이내 실천할 예정 | 6개월 이내 실천할 예정 | 실천할 생각 없음 |
| --- | --- | --- | --- | --- | --- |
| 6) 남을 도울 수 있는 시간 갖기 | ① | ② | ③ | ④ | ⑤ |
| 7) 신앙과 종교 생활하기 | ① | ② | ③ | ④ | ⑤ |

# Part 2. 등교 거부의 욕구-학교에서 느끼는 안전감

문8. 설명을 주의 깊게 읽고, 각 문항이 자신에게 어느 정도 해당된다고 생각하시는지 해당되는 보기를 골라 주십시오.

|  | 전혀 아니다 | 약간 그렇다 | 꽤 그렇다 | 매우 그렇다 |
| --- | --- | --- | --- | --- |
| 학교를 가고 싶지 않을 때가 자주 있다 | ① | ② | ③ | ④ |

문9-11. 학생 주변 환경에 대해 표시(√)하여 주십시오.

|  | 모른다 | 전혀 아니다 | 아니다 | 그렇다 | 매우 그렇다 |
| --- | --- | --- | --- | --- | --- |
| 9)　학교에서는 필요시 누구에게나 도움을 요청할 수 있다 | ① | ② | ③ | ④ | ⑤ |
| 10) 학교 또는 학교 주변 어디가 위험한 장소인줄 알고 있다 | ① | ② | ③ | ④ | ⑤ |
| 11) 학교 주변은 유해환경(술집, 오락실, 게임장 등)으로부터 안 전하다 | ① | ② | ③ | ④ | ⑤ |

# Part 3.지인들로부터의 사회적 지지에 대한 느낌

문.12-17 아래 사람 중 얼마나 쉽게 고민을 털어놓을 수 있나요?

|  | 해당 없음 | 전혀 아니다 | 아니다 | 그렇다 | 매우 그렇다 |
| --- | --- | --- | --- | --- | --- |
| 12) 아버지와 의논할 수 있다 | ① | ② | ③ | ④ | ⑤ |
| 13)　어머니와 의논할 수 있다 | ① | ② | ③ | ④ | ⑤ |
| 14) 형제와 의논할 수 있다 | ① | ② | ③ | ④ | ⑤ |
| 15) 동성 친구와 의논할 수 있다 | ① | ② | ③ | ④ | ⑤ |
| 16) 이성 친구와 의논할 수 있다 | ① | ② | ③ | ④ | ⑤ |
| 17) 학교 선생님과 의논할 수 있다 | ① | ② | ③ | ④ | ⑤ |
